# Effect of pregnancy on serum cytokines in SLE patients

**DOI:** 10.1186/ar3782

**Published:** 2012-03-14

**Authors:** Andrea Doria, Maurizio Cutolo, Anna Ghirardello, Margherita Zen, Danilo Villalta, Angela Tincani, Leonardo Punzi, Luca Iaccarino, Michelle Petri

**Affiliations:** 1 Division of Rheumatology, Department of Clinical and Experimental Medicine, University of Padova, Via Giustiniani 2, Padova, 35128, Italy; 2 Research Laboratory and Academic Unit of Clinical Rheumatology, Department of Internal Medicine, University of Genova, Viale Benedetto XV 6, Genova, 16132, Italy; 3 Hospital S. Maria degli Angeli, Via Montereale 24, Pordenone, 33170, Italy; 4 Rheumatology and Clinical Immunology, Spedali Civili and University of Brescia, Piazza Spedali Civili 1, Brescia, 25123, Italy; 5 Division of Rheumatology and Immunology, Johns Hopkins University School of Medicine, North Wolfe Street 600, Baltimore, MD 21287, USA

## Abstract

**Introduction:**

The aim of this study was to evaluate an extensive panel of cytokines involved in immune regulation during pregnancy in patients with systemic lupus erythematosus (SLE) and in healthy women.

**Methods:**

A total of 47 consecutive successful pregnancies in 46 SLE patients and 56 pregnancies in 56 matched healthy subjects, as controls, were prospectively studied. Serum interleukin (IL)-1-α, IL-1-β, IL-2, IL-6, IL-8, IL-10, IL-12p70, interferon (INF)-γ and tumor necrosis factor (TNF)-α were detected in sera obtained at the first and third trimester of pregnancy by a highly sensitive, multiplexed sandwich ELISA.

**Results:**

Medians (pg/ml) of serum levels of most helper T (Th)1-type cytokines were significantly lower in the third trimester compared with those observed in the first trimester of pregnancy in healthy women: INF-γ 2.0 vs 3.4, TNF-α 10.2 vs 11.5, IL-1-α 0.9 vs 1.1, IL-1-β 0.6 vs 1.0, IL-2 3.0 vs 3.5, and IL-12p70 4.9 vs 5.6 (*P-*values < 0.02 for all). By contrast, only the IL-1-α serum levels were lower in the third trimester compared with the first trimester in SLE patients (*P *= 0.006). IFN-γ/IL-6 and IFN-γ/IL-10 ratios were higher in controls than in SLE (*P *= 0.002, and *P *= 0.001, respectively); moreover, they were significantly reduced in the third compared to the first trimester of pregnancy in healthy women, but not in SLE.

**Conclusions:**

In SLE patients, Th1/Th2 cytokine serum level ratio does not decrease during pregnancy progression as much as in healthy pregnant women. This could account for the observation of a low frequency of disease flares in the third trimester of gestation.

## Introduction

Cytokines play an important role in the pathogenesis of systemic lupus erythematosus (SLE), and their balance seems to affect disease activity [1].

Cytokines produced by CD4+ helper T (Th) cells have been traditionally subdivided into Th1 and Th2 repertoires. The former includes interferon (IFN)-γ, interleukin (IL)-1, IL-2, IL-12, and tumor necrosis factor (TNF)-α, which stimulate cellular immunity, and the latter includes IL-4, IL-5, IL-6 and IL-10, which induce humoral immunity and antibody production [2].

The Th1/Th2 paradigm has recently been overcome by identification of novel Th families, including Th9, Th17 and Th22, with their signature cytokines [3]. In addition, some cytokines considered in the Th1 or Th2 profiles can also be produced by other immune cells. Despite this caveat, the Th1/Th2 cytokine pattern remains an effective, although over-simplistic, model of representing the intricate relationship between immune abnormalities characteristic of autoimmune rheumatic diseases and immune regulatory changes induced by pregnancy.

In SLE, an overproduction of Th2 cytokines, which results in B-cell hyperactivity, has been demonstrated [4] and SLE is thought to be mainly a Th2-driven autoimmune disease. However, abnormalities in Th1 cytokine production are also involved in the pathogenesis of SLE [5].

Pregnancy is a physiological condition during which important changes occur in the maternal immune and endocrine systems [6]. The most important change seems to be the Th2 polarization in maternal immune response, due to a progressive increase in estrogen and progesterone serum levels during pregnancy [7-9].

At high levels, as those achieved during pregnancy, estrogens and progesterone inhibit the secretion of Th1 cytokines, and stimulate the production of Th2 cytokines [6,10]. This Th2 cytokine polarization occurs both at the systemic level and at the fetal-maternal interface [9].

Since SLE is mainly a Th2-mediated disease and during pregnancy an increase in steroid hormone levels occurs, we should expect a subsequent predominance of Th2 response and an exacerbation of the disease, particularly in the third trimester during pregnancy, when estrogens and progesterone levels should be higher [1,11].

However, some studies showed an increased frequency of disease flares in the second trimester and not in the third trimester of pregnancy [1,12-14]. Notably, a lower increase of estrogens, progesterone and Th2 cytokine serum levels in the third trimester of pregnancy in SLE patients compared to that observed in healthy pregnant women has been reported [1,14].

The purpose of this study was to investigate the fluctuations of a large panel of serum cytokines during pregnancy in a considerable number of SLE patients and healthy women, as controls.

## Materials and methods

We prospectively studied 47 successful pregnancies in 46 SLE patients, and 56 pregnancies in 56 healthy women, as controls. All patients fulfilled the 1997 American College of Rheumatology (ACR) classification criteria for SLE [15].

Patients were recruited from SLE cohorts followed at the Departments of Rheumatology of the University of Padova and Brescia (Italy), and at the Johns Hopkins University School of Medicine in Baltimore, Maryland (USA). All subjects declared by written informed consent their approval for study participation, according to the Declaration of Helsinki. The study was approved by the local ethics committee.

The mean age of SLE patients at the time of diagnosis was 23 ± 5.8 years, range 13 to 34 years, and at the time of conception was 30.8 ± 4.4 years, range 18 to 39 years. The mean disease duration at the time of conception was 7.48 ± 5.5 years, range 1 to 20 years. Seven patients were African-American and 39 patients and all healthy controls were Caucasian.

The mean age of healthy controls at the time of conception was 30.2 ± 4.9 years (range 21 to 40 years), matched with that of patients.

During pregnancy and the postpartum period all patients were carefully monitored with monthly rheumatologic and gynecological examinations. Moreover, each patient underwent monthly laboratory analysis as suggested for pregnancy monitoring in SLE [16].

Disease activity was assessed before pregnancy according to ECLAM (European Consensus Lupus Activity Measurement) [17] and during pregnancy with ECLAM modified for pregnancy [18]. SLE was considered active in the case of ECLAM score ≥ 2.

### Determination of cytokines in sera

Serum samples for cytokine assays were collected respectively at the first (9 to 11 weeks) and third (29 to 31 weeks) trimester of gestation, and stored in aliquots at -80°C until analyses were performed.

Serum levels of IL-1-α, IL-1-β, IL-2, IL-6, IL-8, IL-10, IL-12p70, IFN-γ and TNF-α were quantitatively determined by a sandwich highly sensitive enzyme-linked immunosorbent assay (ELISA) designed with multiplex technology (SearchLight Human Inflammatory Cytokine Array by Pierce Biotechnology, Rockford, IL, USA) [19]. In 2006, this commercial kit was approved by the National Institute of Health in the United States (U.S. NIH) as a reference method for the quantitative determination of cytokines in human biological fluids.

The assay procedure was performed according to the manufacturer's instructions.

The ranges of measurability of the standard curve for each cytokine tested were: IL-1-α = 0.78 to 200 pg/ml; IL-1-β = 0.39 to 100 pg/ml; IL-2 = 0.78 to 200 pg/ml; IL-6 = 0.78 to 200 pg/ml; IL-8 = 0.78 to 200 pg/ml; IL-10 = 0.78 to 200 pg/ml; IL-12p70 = 1.2 to 300 pg/ml; IFN-γ = 0.78 to 200 pg/ml, TNF-α = 4.7 to 1,200 pg/ml.

The analytic sensitivity was: IL-1-α = 0.4 pg/ml; IL-1-β = 0.2 pg/ml; IL-2 = 0.4 pg/ml; IL-6 = 0.2 pg/ml; IL-8 = 0.4 pg/ml; IL-10 = 0.2 pg/ml; IL-12p70 = 0.4 pg/ml; IFN-γ = 0.2 pg/ml; TNF-α = 1.6 pg/ml.

### Statistical analysis

Statistical analysis was performed with the SPSS 16.0 program (Bologna, Italy) using the Wilcoxon test for paired data and the Mann-Whitney U test.

The choice of a non-parametric statistical analysis was due to the fact that the values of cytokine serum levels did not follow normal distribution, both in healthy controls and in SLE patients.

We applied the Mann-Whitney U test to compare cytokine median levels measured in patients and in healthy women in the first and the third trimester of pregnancy. We corrected the Mann-Whitney univariate analysis by using the Bonferroni test for multiple comparisons, considering a significant *P-*value of 0.0056.

To analyze the longitudinal changes in cytokine levels during pregnancy, we used the Wilcoxon test for paired data. The graphical representation of the results was carried out by the so-called box plots displaying the median, the range of values between the 25^th ^and the 75^th ^percentiles, and the range of values obtained. We considered as significant a *P-*value < 0.05.

## Results

As stated in the inclusion criteria, all pregnancies successfully ended with the birth of a living baby. There were no differences between the mean weight of babies born from SLE women and healthy controls. The duration of gestation was similar in SLE patients (37.5 weeks, range 27.3 to 45.7) and in controls (38.1 weeks, range 27.5 to 44.8 weeks).

The ECLAM score before pregnancy ranged from 0 to 4.5, (mean ± SD 1.86 ± 1.21, median value 2). At the time of conception the disease was in remission in 30 patients (65%), while it was still active in the remaining 16 patients (35%). In these patients, hematological and joint involvements were the most frequent manifestations. Moreover, 16 patients (34.1%) had renal involvement at the time of conception. Clinical and laboratory features of the 46 patients at the time of conception are summarized in Table 1.

**Table 1:** Clinical and laboratory features at conception and treatment in 46 SLE patients during 47 pregnancies.

	No. of patients (%)
**Skin manifestations**	16 (35)
**Arthralgias and/or arthritis**	26 (56)
**Serositis**	4 (8.7)
**Glomerulonephritis**	16 (34)
**CNS involvement**	2 (4.3)
**Hematological involvement**	26 (56)
**Anti-dsDNA Ab**	33 (71.7)
**Anti-Sm Ab**	6 (13)
**Anti-U1RNP Ab**	10 (21.7)
**Anti-Ro/SSA Ab**	17 (37)
**Anticardiolipin Ab**	20 (43.5)
**LAC**	7 (15.2)
**Double LAC + aCL positivity**	6 (13)
**Prednisone**	36 (78)
**Antimalarials**	18 (39)
**Azathioprine**	11 (24)
**No therapy**	5 (11)

Hematological involvement, excluding hemolytic anemia. Ab, anti-extractable nuclear antigens antibodies (anti-ENA); aCL, anticardiolipin antibodies Anti-dsDNA Ab, anti-double-stranded-DNA antibodies; anti-Sm, anti-U1RNP, anti-Ro/SSA; CNS, central nervous system; LAC, lupus anticoagulant

During pregnancy 36 patients (76.5%) had received corticosteroid therapy at the mean dose of 10.56 ± 9.3 mg/day (range 2.5 to 40) in the first trimester of pregnancy, 10.72 ± 9.27 mg/day (range 2.5 to 40) in the second trimester, and 10.71 ± 8.8 mg/day (range 2.5 to 40) in the third trimester; in addition, 18 patients (38.3%) had received an anti-malarial (hydroxychloroquine), 11 (23.4%) azathioprine, and 5 (10.6%) were free of therapy during pregnancy.

Patients with active SLE received a median dose of prednisone higher than patients with inactive disease: 12.3 mg/day (range 5 to 40) vs. 4.6 mg/day (range 2.5 to 12.5) (*P *= 0.008).

We did not observe differences in treatment, disease activity and pregnancy outcome in African-American patients compared to Caucasian SLE patients.

Sixteen of the 46 SLE patients fulfilled the criteria for anti-phospholipid syndrome (APS): 14 had previous miscarriages, and 2 had previous thrombotic events. None of the patients had thrombotic events during this study.

We observed three cases of pre-eclampsia: two in patients with a history of renal involvement before pregnancy but with SLE in remission at the time of conception, treated with low dose prednisone ( < 10 mg/day), while the third case in a patient with active glomerulonephritis at the time of conception, treated with high dose prednisone ( > 10 mg/day) in combination with azathioprine and anti-malarial.

### Cytokines

The cytokine median levels in the first and third trimesters of pregnancy in healthy women and in SLE patients are reported in Table 2.

**Table 2:** Th1 and Th2 cytokine levels during pregnancy in 56 healthy women and 46 SLE patients

	Healthy women			SLE patients		
	**1^st ^trimester**	**3^rd ^trimester**	***P *=**	**1^st ^trimester**	**3^rd ^trimester**	***P *=**
**TNF-α**	11.5 (1.4 to 104.6)	10.2 (0.7 to 72.9)	0.001	13.5 (0 to 210.5)	11.0 (0 to 174.5)	n.s.
**IFN-γ**	3.4 (0 to 29.0)	2.0 (0.1 to 55.5)	0.001	2.8 (0 to 75.0)	3.0 (0 to 38.2)	n.s.
**IL-1α**	1.1 (0 to 13.2)	0.9 (0 to 12.8)	0.003	1.0 (0 to 56.6)	1.0 (0 to 27.5)	0.006
**IL-1β**	1.0 (0 to 10.3)	0.6 (0 to 70.8)	0.018	0.8 (0 to 35.2)	0.5 (0 to 18.4)	n.s.
**IL-2**	3.5 (0 to 39.8)	3.0 (0 to 206)	0.018	4.5 (0 to 122.4)	4.1 (0 to 40.3)	n.s.
**IL-8**	5.2 (0.9 to 112.6)	4.4 (0.9 to 145.9)	0.053	7.4 (0 to 910.9)	4.6 (0 to 717.1)	n.s.
**IL-12**	5.6 (0.1 to 70.6)	4.9 (0 to 29.4)	0.018	6.0 (0 to 186.5)	4.2 (0 to 105.7)	n.s.
**IL-10**	1.3 (0 to 45.0)	1.1 (0 to 19.1)	n.s.	2.9 (0 to 124.1)	3.1 (0 to 73.6)	n.s.
**IL-6**	5.5 (1.8 to 10.4)	4.8 (2.2 to 9.2)	n.s.	6.3 (3.5 to 22.6)	7.3 (3.7 to 23.1)	n.s.

IFN-γ, interferon gamma; IL, interleukin; n.s., not significant; TNF-α, tumor necrosis factor alpha. The median values (25^th ^to 75^th ^percentile) are expressed in pg/ml. Differences between the median cytokine serum levels in the first and third trimester were statistically evaluated by Wilcoxon test for paired data (significant *P-*value < 0.05).

In healthy women, Th1-type cytokine serum levels showed a significant reduction in the third trimester versus the first trimester of gestation while no significant changes were observed in IL-6 and IL-10 serum levels between the first and third trimester of pregnancy.

In SLE patients, IL-1-α was the only Th1 cytokine whose levels were significantly decreased in the third trimester compared to those observed in the first trimester (*P *= 0.006). Serum levels of all the other Th1 cytokines tested and Th2 cytokines IL-6 and IL-10 did not significantly change between the first and the third trimester of pregnancy.

**Figure 1:**
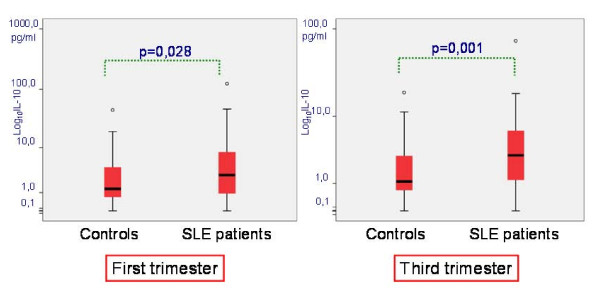
**IL-10 levels in the first and third trimesters of pregnancy in controls and SLE patients**.

IL-10 serum levels were higher in SLE patients compared to controls in the third trimester of pregnancy (*P *= 0.001) (Figure 1). Moreover, in the third trimester the difference in IL-10 serum levels between active SLE and controls (*P *= 0.004) was greater than between inactive SLE and controls (*P *= 0.015, which is not significant when corrected by Bonferroni test multiple comparisons).

We did not observe any difference in cytokine levels in African-American SLE patients compared to Caucasian patients.

Both in SLE patients and in healthy women cytokine, serum levels did not correlate with age at the time of conception.

In the first trimester, IL-2, IL-12, IFN-γ and IL-6 serum levels correlated with ECLAM score (IL-2 r = 0.524 *P *= 0.005, IL-12 r = 0.549 *P *= 0.007, IFN-γ r = 0.492 *P *= 0.017, IL-6 r = 0.515 *P *= 0.02) in SLE women.

The median (25^th ^to 75^th ^percentiles) cytokine serum levels were similar in patients with and without glomerulonephritis both in the first and in the third trimester of pregnancy (Table 3).

**Table 3:** Cytokine levels during the first and third trimester in SLE patients with/without glomerulonephritis before pregnancy

	1^st ^trimester			3^rd ^trimester		
	SLE patients with glomerulonephritis	SLE patients without glomerulonephritis	*P*	SLE patients with glomerulonephritis	SLE patients without glomerulonephritis	*P*
TNF-α	14.90 (6.50 to 41.00)	10.90 (5.30 to 25.82)	n.s.	25.90 (8.20 to 38.80)	10.10 (5.97 to 30.25)	n.s.
INF-γ	4.40 (2.20 to 13.00)	3.20 (0.25 to 4.67)	n.s.	5.60 (1.50 to 15.80)	2.95 (1.52 to 6.95)	n.s.
IL-1α	3.00 (0.50 to 10.00)	0.85 (0.27 to 1.37)	n.s.	1.50 (0.40 to 6.60)	1.10 (0.12 to 2.20)	n.s.
IL-1β	1.10 (0.37 to 10.025)	1.95 (1.15 to 4.30)	n.s.	0.70 (0.32 to 3.37	1.80 (0.60 to 9.30)	n.s.
IL-2	6.20 (1.50 to 15.20)	2.15 (1.57 to 4.05)	n.s.	6.50 (1.10 to 12.80)	2.50 (1.35 to 6.12)	n.s.
IL-8	8.75 (6.70 to 40.75)	27.15 (8.72 to 52.37)	n.s.	7.40 (4.70 to 29.30)	24.85 (4.22 to 197.40)	n.s.
IL-12	6.40 (2.00 to 22.70)	4.05 (1.77 to 18.02)	n.s.	4.20 (1.70 to 25.50)	3.30 (2.02 to 20.65)	n.s.
IL-10	3.25 (0.90 to 12.12)	2.50 (1.95 to 4.40)	n.s.	4.20 (1.55 to 11.65)	4.60 (2.50 to 5.80)	n.s.
IL-6	8.10 (3.50 to 38.10)	5.50 (2.90 to 6.50)	n.s.	11.25 (1.55 to 25.57)	9.40 (5.20 to 12.90)	n.s.

SLE patients with glomerulonephritis: *n *= 16; SLE patient without glomerulonephritis: *n *= 30. The median values (25^th ^to 75^th ^percentile) are expressed in pg/ml.

There were no significant differences in cytokine serum levels among the 27 patients receiving low-dose steroid therapy ( ≤ 10 mg/day) and the 9 patients taking steroids at higher dose ( > 10 mg/day) in both the first and the third trimester of gestation (Table 4).

**Table 4:** Cytokine levels in SLE patients according to steroids intake during the first and third trimester.

	1^st ^trimester			3^rd ^trimester		
	High dose PDN	Low dose PDN	*P*	High dose PDN	Low dose PDN	*P*
TNF-α	21.70 (5.75 to 64.75)	10.70 (6.50 to 38.32)	n.s.	18.50 (5.25 to 34.75)	10.25 (5.05 to 25.45)	n.s.
INF-γ	4.80 (1.15 to 15.30)	3.75 (1.32 to 11.62)	n.s.	8.20 (0.40 to 14.05)	3.40 (1.12 to 10.00)	n.s.
IL-1α	0.50 (0.15 to 5.70)	1.90 (0.50 to 7.90)	n.s.	1.20 (0.0 to 2.85)	1.30 (0.25 to 5.95)	n.s.
IL-1β	0.80 (0.25 to 2.05)	1.30 (0.45 to 4.20)	n.s.	0.10 (0.0 to 5.80)	0.90 (0.15 to 2.40)	n.s.
IL-2	6.00 (3.10 to 14.00)	3.55 (1.50 to 13.92)	n.s.	4.80 (1.90 to 13.45)	4.40 (1.50 to 11.90)	n.s.
IL-8	8.70 (2.55 to 25.65)	8.65 (4.62 to 33.70)	n.s.	3.90 (0.50 to 6.40)	4.70 (2.10 to 19.80)	n.s.
IL-12	6.40 (3.85 to 20.05)	8.35 (1.87 to 31.60)	n.s.	3.20 (0.50 to 21.30)	4.45 (2.12 to 24.97)	n.s.
IL-10	2.10 (1.20 to 8.45)	3.60 (1.22 to 13.57)	n.s.	2.00 (1.35 to 9.50)	3.90 (1.37 to 9.67)	n.s.
IL-6	6.50 (4.45 to 17.75)	8.20 (3.87 to 34.55)	n.s.	6.30 (4.05 to 29.90)	7.70 (3.90 to 25.50)	n.s.

SLE patients who have taken high steroids dose: *n *= 27; SLE patients who have taken low steroids dose: 9. The median values (25^th ^to 75^th ^percentile) are expressed in pg/ml.

Moreover, no differences in cytokine levels among patients treated with or without anti-malarial were observed.

No differences in cytokine serum levels in SLE patients with a history of pregnancy loss compared with those without were observed; moreover, cytokine levels did not correlate with the number of previous pregnancy losses.

**Figure 2:**
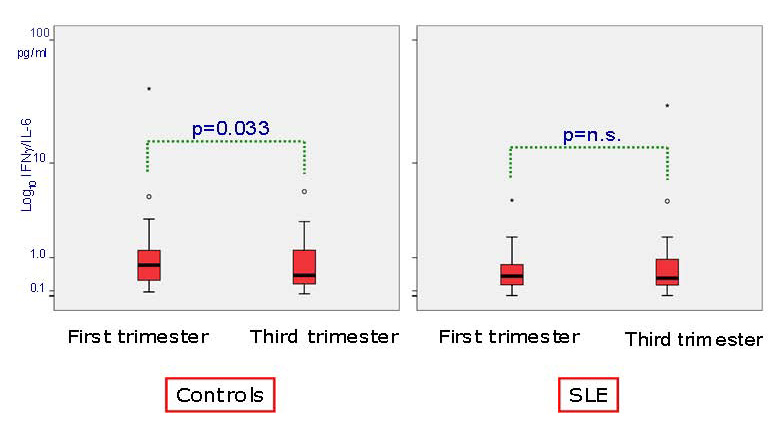
**IFNγ/IL-6 ratio in controls and SLE patients: comparison between first and third trimesters of pregnancy**.

The ratio between IFN-γ and IL-6 serum levels (IFN-γ/IL-6) was significantly lower in the third compared to the first trimester (median 0.43, range 0.04 to 220.20, vs 0.71, range 0.08 to 41.00, *P *= 0.033) in healthy controls, but not in SLE patients (median 0.34, range 0.01 to 30.00, vs 0.43, range 0.01 to 4.63, *P *= n.s.) (Figure 2).

The ratio between IFN-γ and IL-10 serum levels (IFN-γ/IL-10) was higher in healthy women compared to SLE patients in both the first (median 1.88, range 1.30 to 3.67, vs 0.97, range 0.44 to 1.93, *P *= 0.001) and the third trimester of pregnancy (median 2.00, range 0.90 to 3.10, vs 0.83, range 0.35 to 1.48, *P *= 0.001).

## Discussion

The increase in cortisol, progesterone and estradiol concentrations observed during pregnancy in healthy women has been associated with an increase in cytokines predominantly produced by Th2 cells and a reduction in those mainly produced by Th1 cells [1,20]. It has been shown that estrogens and progesterone are able to modulate antibody production [21], by promoting Th2 polarization. In fact, estrogens at pharmacological levels seem to stimulate antibody production by increasing IL-6 and IL-10 secretion [22,23], whereas high levels of progesterone, such as those observed in the second part of gestation, seem to stimulate the production of IL-4, IL-5 and IL-10 [24] and inhibit Th1 type cytokine production [6].

IL-6 is a pleyotropic cytokine that plays an important role in the differentiation of Th0 cells toward a Th2 phenotype, inhibiting the development of a Th1-mediated response [25]. It has been shown that SLE patients have elevated IL-6 serum levels which correlate with disease activity or anti-dsDNA antibodies [26,27]. IL-6 promotes the proliferation of renal mesangial cells in mice and several studies report IL-6 as a critical mediator of tissue damage due to its ability to stimulate the production of nephritogenic antibodies [28].

In SLE patients, IL-10 is also overproduced [29-32] and IL-10 serum concentration correlates with disease activity [26,33,34]. IL-10 is a powerful stimulator of B cell proliferation, differentiation and antibody secretion [35], as well as an inhibitor of antigen presenting cell and T cell functions [4,36]. IL-10 may be also influenced by the use of glucocorticoids. However, in our study no differences in IL-10 serum levels between patients taking steroids at high or low dose were observed.

The role of Th1 cytokines in the pathogenesis of SLE is less clear and probably systemic and local effects are different. It has been demonstrated that TNF-α leads to T cell hypo-responsiveness and controls autoreactivity inducing T cell apoptosis in the peripheral blood [37]. On the other hand, TNF-α has been found in kidney and skin biopsies in SLE and is thought to be responsible for tissue damage [38,39].

Since the activation of the Th2 cytokines seems to be a relevant pathway in the pathogenesis of SLE, the gestation-induced Th2 polarization of immune response might theoretically contribute to a disease flare during pregnancy in SLE women [1,11].

In our study, IL-6 and TNF-α serum levels were similar in SLE patients and controls and only the IL-10 level was higher in patients than in controls. The most relevant feature in our study was a lower decrease of Th1 cytokine and a lower increase of Th2 cytokine serum levels in SLE pregnant women compared with healthy pregnant controls. This profile is confirmed by the lower than expected reduction of IFNγ/IL-6 ratio (Figure 2) in the third trimester of SLE pregnancy.

In our previous study, we demonstrated that IL-6 serum levels were lower in pregnant SLE patients than in healthy pregnant controls in the third trimester of pregnancy; by contrast, IL-10 serum levels in SLE pregnant patients were higher during the whole pregnancy [1].

Moreover, our data appear similar to those recently published by Torricelli *et al. *[40]. In their study, they also found higher levels of IL-10 in pregnant patients affected with SLE compared with healthy pregnant women. Interestingly, in SLE patients IL-10 level progressively increased during pregnancy.

Furthermore, another study [41] showed higher levels of IL-10 in pregnant patients affected with SLE than in healthy pregnant women, particularly during the third trimester of pregnancy. In this study, IL-6 was not measured.

Thus, a lower Th2 polarization was observed during pregnancy in SLE patients compared with healthy pregnant controls, especially in the third trimester of gestation. This feature may be explained by the lower than expected levels of estrogen and progesterone during the second and third trimester of pregnancy, which we have recently reported [14]. The lower Th2 polarization during pregnancy could account for the lack of differences in disease flare between pregnant and non-pregnant SLE patients reported in some controlled studies [12,42,43].

Since Th2 polarization is thought to be important for the trophoblast to invade and anchor to the deciduas, ensuring pregnancy maintenance [44] one may argue why our patients were able to successfully complete their pregnancies. A potential explanation is that cytokine patterns could be different at the feto-maternal interface compared with those measured in the systemic circulation, and the low levels of estrogen and progesterone produced by the placenta could at least be sufficient to induce a localized Th2 polarization at this level, allowing a maternal tolerance to paternal antigens and thus a successful gestation.

A further intriguing aspect in this field is the trimester of pregnancy which is at higher risk for a disease relapse. Data on the timing of disease flares during pregnancy are conflicting. Since during pregnancy the Th2 immune deviation is primarily due to the increase in estrogen and progesterone levels, exacerbations of disease should be expected more frequently during the third trimester, when these hormones reach their highest levels. By contrast, recent studies report a lower number of relapses in the third trimester than in the second trimester of gestation or post partum [1,12-14,43,45-48]. This variation may also be explained by the different Th1/Th2 balance observed in SLE pregnant women, compared with healthy pregnant controls caused by the lower estrogen and progesterone levels observed in SLE patients during the third trimester.

## Conclusions

Our study demonstrates that the induction of a predominant Th2 cytokine milieu during pregnancy is less evident in SLE patients than in healthy pregnant women. This cytokine profile, characterized by a lower Th2 response could explain why, in some studies, SLE flares are not more common in pregnant than in non-pregnant patients and why, if they occur, they rarely take place during the third trimester of gestation.

## Abbreviations

Ab: antibody; aCL: anticardiolipin antibodies; ACR: American College of Rheumatology; APS: antiphospholipid syndrome; CNS: central nervous system; dsDNA: double stranded DNA; ECLAM: European Consensus Lupus Activity Measurement; ELISA: enzyme linked immunosorbent assay; ENA: extractable nuclear antigens; IL: interleukin; INF: interferon; LAC: lupus anticoagulant; PDN: prednisone; pg/ml: pycogram/milliliter; SD: standard deviation; SLE: systemic lupus erythematosus; Th: T helper; TNF: tumor necrosis factor; U.S. NIH: National Institute of Health in the United States.

## Competing interests

The authors declare that they have no competing interests.

## Authors' contributions

AD conceived the study, participated in its design and coordination, helped to draft the manuscript and is responsible for the final version. MC gave helpful suggestions to the study design and critically revised the manuscript. AG and DV carried out the cytokine dosage and looked after data collection. AG also revised the manuscript. LI and MZ followed up patients, participated in the design of the study and drafted the manuscript. MP took care of patients from the United States, participated in the design of the study and revised the manuscript. AT followed up patients. LP revised the manuscript. All authors read and approved the final manuscript.
